# The Impact of DAZZEON αSleep^®^ Far-Infrared Blanket on Sleep, Blood Pressure, Vascular Health, Muscle Function, Inflammation, and Fatigue

**DOI:** 10.3390/clockssleep6030033

**Published:** 2024-09-04

**Authors:** Mon-Chien Lee, Chin-Shan Ho, Yi-Ju Hsu, Nai-Wen Kan, Chen-Yin Fei, Hung-Jen Yang, Chi-Chang Huang

**Affiliations:** 1 Graduate Institute of Sports Science, National Taiwan Sport University, Taoyuan City 333325, Taiwan; 1061304@ntsu.edu.tw (M.-C.L.); kilmur23@ntsu.edu.tw (C.-S.H.); ruby780202@ntsu.edu.tw (Y.-J.H.); 2 Center for General Education, Taipei Medical University, Taipei City 110301, Taiwan; kevinkan@tmu.edu.tw; 3 Dazzeon Biotech Co., Ltd., New Taipei City 248022, Taiwan; elyse.fei@gmail.com; 4 Department of General Medicine, Min-Sheng General Hospital, Taoyuan City 330063, Taiwan; 5 Tajen University, Pingtung County 907101, Taiwan

**Keywords:** far-infrared radiation, blanket, sleep, fatigue, vascular health

## Abstract

The application of far-infrared blankets has shown certain benefits in health promotion and therapy, such as improving blood circulation and alleviating muscle pain. However, the effects of such blankets on increasing deep sleep, reducing blood pressure, enhancing memory, dilating microvessels for blood flow, reducing chronic inflammation, and decreasing fatigue remain to be studied. We aim to investigate the effects of the DAZZEON αSleep^®^ far-infrared blanket on these indicators. This study adopted a double-blind design, recruiting 24 male participants aged over 45 years, divided into two groups of 12 each: (A) a placebo group and (B) a DAZZEON αSleep^®^ group. The participants used the blanket every night for two weeks, with sleep records taken using a wearable device and blood pressure, blood oxygen levels, arterial stiffness, and surface temperature measured before and after the intervention. Blood samples were collected for an analysis of inflammation and sleep-related blood indicators (serotonin and melatonin), and exercise tests were conducted to assess fatigue improvement. Compared with before the intervention, the blanket significantly increased changes in grip strength and reaction time. Additionally, it significantly increased blood serotonin, melatonin, and nitric oxide concentrations (*p* < 0.05), thus significantly increasing deep sleep and REM sleep durations (*p* < 0.05) and improving subjective sleep quality (*p* < 0.05). This study confirmed that using the DAZZEON αSleep^®^ far-infrared blanket for 14 consecutive days helps to improve blood circulation, reduce vascular age and arterial stiffness, increase serotonin and melatonin levels, and improve sleep quality, as well as enhances muscle strength and reaction time.

## 1. Introduction

Far-infrared (FIR) radiation, characterized by wavelengths of 3 to 1000 µm, has garnered significant attention in therapeutic contexts due to its ability to penetrate deeply into human tissue, offering a range of health benefits such as improved circulation, muscle relaxation, and enhanced detoxification processes. The biophysical mechanisms underlying these benefits are attributed to FIR-induced changes in cellular function, which include increased microcirculation and enhanced metabolic rates [1]. These properties make FIR an appealing non-pharmacological treatment modality, particularly valued for its capacity to promote beneficial biological processes without adverse effects [2].

Recent advancements have facilitated the integration of FIR-emitting materials into textiles, creating garments that deliver these therapeutic effects continuously, which is an innovative approach that avoids the need for an active intervention or direct supervision [2]. This development has had significant implications in both clinical and athletic settings. In sports, for instance, FIR textiles have been shown to enhance athletic performance and recovery by improving muscle oxygenation and reducing fatigue. FIR is thought to be effective in accelerating recovery from muscle damage caused by eccentric exercise [3]. A recent study in 2024 highlighted how FIR radiation lamp therapy substantially aided in recovery following a simulated soccer match among elite female soccer players, showcasing improvements in muscle soreness and systemic recovery [4].

In medical settings, particularly for patients with chronic conditions such as diabetes mellitus, FIR textiles have demonstrated potential in improving peripheral blood flow and oxygenation. These factors are crucial in managing and preventing complications such as diabetic foot ulcers [1]. Furthermore, the impact of FIR on sleep quality has been explored, with studies indicating that FIR-emitting pajamas can enhance sleep efficiency and reduce awakening time, underscoring the technology’s role in promoting better sleep and overall health [5]. This may be because far-infrared rays can eliminate toxins by stimulating the vibrational resonance of water molecules. Based on this phenomenon, an increase in molecular kinetic energy leads to an increase in skin and muscle temperature and vasodilation [6]. Previous research has found that far-infrared fiber can enhance metabolic rate, blood oxygen levels, and blood circulation and eliminate metabolic toxins [7,8]. In addition, far-infrared rays have thermal therapeutic effects because they are absorbed by skin tissue. These warming effects are known to increase core body temperature, thereby promoting muscle relaxation and blood circulation, as well as activating metabolism [9].

The broader applications of FIR for enhancing well-being and reducing stress were evidenced in a 2023 crossover trial that assessed the effects of FIR heaters on autonomic nervous activity and mood states. The study found that exposure to FIR, especially of the feet, positively influenced mood states and autonomic nervous function, suggesting potential applications in mental health and stress reduction [10].

This research aims to build upon these findings by examining the continuous use of the DAZZEON αSleep^®^ far-infrared blanket over a 14-day period, focusing on its effects on body composition, physical performance, and sleep quality. In addition, we not only assess the health status of and changes displayed in the subjects through blood biochemical analysis but also analyze sleep-related blood indicators to understand whether the device can help promote blood circulation and improve blood sleep-related indexes, which serves as the basis for further explorations of its mechanisms. By doing so, this study seeks to substantiate the potential use of FIR as a preventive and therapeutic measure in both wellness and clinical contexts, thereby contributing significantly to the existing literature on the therapeutic benefits of far-infrared technologies.

## 2. Results

### 2.1. Changes in Body Composition before and after Using the DAZZEON αSleep^®^ Far-Infrared Blanket

As shown on Table 1, there were no significant differences between the placebo and DAZZEON αSleep^®^ groups in terms of SpO_2_, body weight, BMI muscle mass, or fat mass in the pre- and post-tests, respectively, and there was no significant change within each group.

### 2.2. Changes in Surface Temperature before and after Using the DAZZEON αSleep^®^ Far-Infrared Blanket

The participants’ bodies were divided into three regions for thermal imaging: (a) the upper body (from the top of the head to the thymus), (b) the chest (from the thymus to the pelvis), and (c) the legs (from the pelvis to the soles of the feet). The highest temperature was recorded. The temperature changes from before to after covering with the blanket were measured before and after the intervention for 14 consecutive days. As shown in Table 2, the main effect of group was significant (F(7, 104) = 5.274, *p* = 0.024, η^2^ = 0.048), indicating that there was a significant difference between the DAZZEON αSleep^®^ group and the placebo group. Specifically, the mean temperature of the DAZZEON αSleep^®^ group was significantly higher than the mean temperature of the placebo group. The main effect when comparing the before and after measurements was significant (F(7, 104) = 13.679, *p* < 0.001, η^2^ = 0.116), indicating that there was a significant difference between the two measurements. Specifically, the mean after-measurement temperature was significantly higher than the mean before-measurement temperature.

### 2.3. Effects of the DAZZEON αSleep^®^ Far-Infrared Blanket on Vascular Age and Arteriosclerosis

In this study, there were no significant main effects or interactions between the groups (the placebo and DAZZEON αSleep^®^ groups), times (before and after), or before and after measurements (before and after) of the vascular age and right and left baPWV (Table 3).

### 2.4. Effects of the DAZZEON αSleep^®^ Far-Infrared Blanket on Muscle Elasticity and Hardness

As shown in Table 4, the time × measurement effect on the decrease in the right biceps brachii was significant (F(7, 104) = 4.147, *p* = 0.044, η^2^ = 0.038). However, through post hoc analysis, comparing before with after via an examination of the pre- and post-time points, it was found that there was no significant difference. The group × time effect on frequency (F(7, 104) = 4.456, *p* = 0.037, η^2^ = 0.041) and stiffness (F(7, 104) = 5.563, *p* = 0.020, η^2^ = 0.051) of the left biceps brachii was significant. In addition, after the 14-day intervention, the DAZZEON αSleep^®^ group showed significantly greater results than before the intervention in terms of frequency (*p* = 0.018) and stiffness (*p* = 0.011), assessed using post hoc analysis. The left rectus femoris had a significant group effect on frequency (F(7, 104) = 21.505, *p* < 0.001, η^2^ = 0.171) and stiffness (F(7, 104) = 5.357, *p* = 0.023, η^2^ = 0.049), and it had a significant time effect on frequency (F(7, 104) = 14.032, *p* < 0.001, η^2^ = 0.121) and stiffness (F(7, 104) = 6.597, *p* = 0.012, η^2^ = 0.060).

### 2.5. Effects of the DAZZEON αSleep^®^ Far-Infrared Blanket on Muscle Strength, Cardiopulmonary Endurance, and Agility

**Figure 1:**
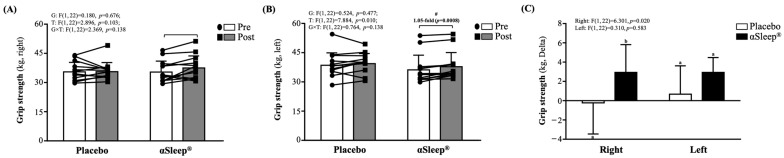
Effects of 2-week DAZZEON αSleep^®^ intervention on (**A**) right-hand grip strength, (**B**) left-hand grip strength, and (**C**) delta pre–post hand grip strength. Data are expressed as mean ± SD. Different superscript letters (a, b) indicate significant differences at *p* < 0.05 at the same time point. # indicates a significant effect (*p* < 0.05) after 2-week intervention compared with before intervention.

Both before and after the intervention, there were no significant differences between the placebo and DAZZEON αSleep^®^ groups. However, after 14 consecutive days of the intervention, only the DAZZEON αSleep^®^ group showed a significant increase in left-hand (*p* = 0.008) grip strength compared to before the intervention (Figure 1B). There was a significant time effect (F(1, 22) = 7.884, *p* = 0.010, η^2^ = 0.231). In addition, the delta before and after scores of right-hand grip strength in the DAZZEON αSleep^®^ group were significantly greater than in the placebo group (F(1, 22) = 6.301, *p* = 0.020) (Figure 1C).

**Figure 2:**
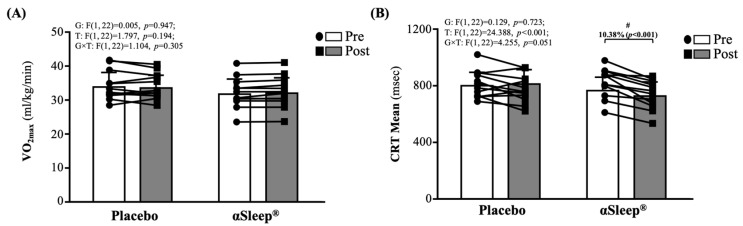
Effects of 2-week DAZZEON αSleep^®^ intervention on (**A**) VO_2max_ and (**B**) complex reaction time assessed using Fitlight test. Data are expressed as mean  ±  SD. # indicates a significant effect (*p* < 0.05) after 2-week intervention compared with before intervention.

As shown in Figure 2A, there were no significant differences between the placebo and DAZZEON αSleep^®^ groups before and after the 14-day intervention. As shown in Figure 2B, there were no significant differences between the placebo and DAZZEON αSleep^®^ groups before the intervention. However, after the 14-day intervention, the DAZZEON αSleep^®^ group showed a significantly reduced CRT mean, reduced by 10.38% (*p* < 0.001), and there was a significant time effect (F(1, 22) = 24.388, *p* < 0.001, η^2^ = 0.526).

### 2.6. Effects of the DAZZEON αSleep^®^ Far-Infrared Blanket on Blood Biochemistry

All participants underwent blood parameter analysis before and after the intervention to determine their physiological status. This was carried out to ensure that the 14-day intervention did not result in any adverse side effects. The study confirmed that all subjects were in good health during the trial, and there were no significant differences in various blood indicators measured between the placebo group and DAZZEON αSleep^®^ group. However, after the 14-day intervention, the DAZZEON αSleep^®^ group showed a significantly reduced TG level (*p* = 0.039) and significantly increased HDL level (*p* = 0.005) compared to before the intervention (Table 5). Both TG (F(1, 22) = 5.894, *p* = 0.024, η^2^ = 0.211) and HDL (F(1, 22) = 7.538, *p* = 0.012, η^2^ = 0.255) concentrations showed significant group × time interactions.

**Figure 3:**
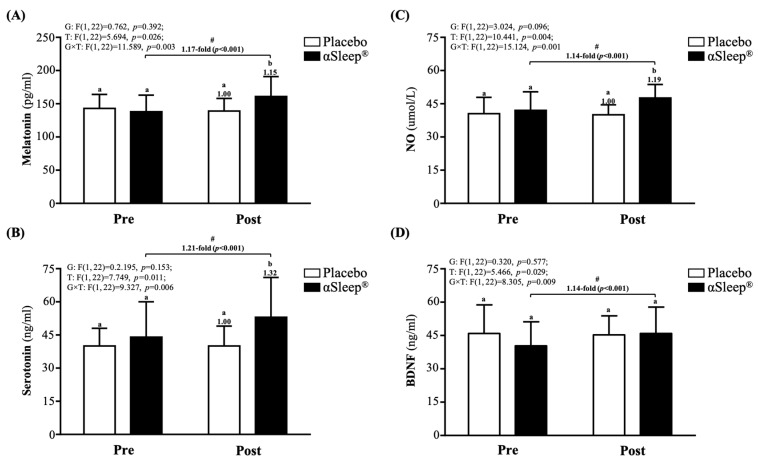
Effects of 2-week DAZZEON αSleep^®^ intervention on (**A**) melatonin, (**B**) serotonin, (**C**) NO, and (**D**) BDNF. Data are expressed as mean ± SD. Different superscript letters (a, b) indicate significant differences at *p* < 0.05 at the same time point. # indicates a significant effect (*p* < 0.05) after 2-week intervention compared with before intervention.

After the 14-day intervention, the DAZZEON αSleep^®^ group showed a significantly increased 1.15-fold melatonin level (F(1, 22) = 4.366, *p* = 0.048) (Figure 3A), 1.32-fold increased serotonin level (F(1, 22) = 4.742, *p* = 0.040) (Figure 3B), and 1.19-fold increased NO level (F(1, 22) = 12.228, *p* = 0.039) (Figure 3C) compared with the placebo group. The melatonin, serotonin, NO, and BDNF levels were significantly increased compared to before the intervention (*p* < 0.05) in the DAZZEON αSleep^®^ group (*p* < 0.05), and there were significant time and group × time effects (F(1, 22) = 5.466 to 15.124, *p* < 0.05, η^2^ = 0.206 to 0.345).

### 2.7. Effects of the DAZZEON αSleep^®^ Far-Infrared Blanket on Complete Blood Count

As shown in Table S1, there were no significant differences in all test indicators between the placebo group and the DAZZEON αSleep^®^ group from before the intervention to 14 days after the intervention. In addition, there were no significant changes as determined in a within-group comparison of the two groups.

### 2.8. Effects of the DAZZEON αSleep^®^ Far-Infrared Blanket on Subjective Sleep Quality (Pittsburgh Sleep Quality Index, PSQI)

**Figure 4:**
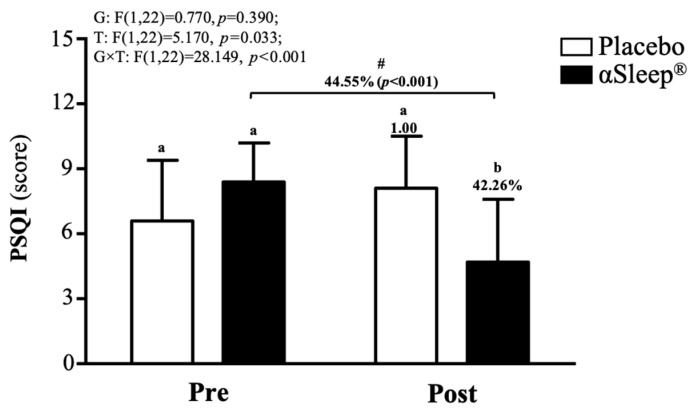
Effects of 2-week DAZZEON αSleep^®^ intervention on PSQI score. Data are expressed as mean  ±  SD. Different superscript letters (a, b) indicate significant differences at *p* < 0.05 at the same time point. # indicates a significant effect (*p* < 0.05) after 2-week intervention compared with before intervention.

The subjects’ sleep outcomes are shown in Table S2; there were no significant differences between the placebo group and DAZZEON αSleep^®^ group in bed time, time to fall asleep, wake time, or total sleep time. According to the PSQI score, before the intervention, there were no significant differences between the placebo and DAZZEON αSleep^®^ groups. However, after the 14-day intervention, the DAZZEON αSleep^®^ group showed a significantly reduced PSQI score, reduced by 42.27% (F(1, 22) = 9.904, *p* = 0.005). In addition, only the DAZZEON αSleep^®^ group showed a significantly reduced PSQI score compared to before the intervention (*p* < 0.001). The PSQI score showed a significant time effect (F(1, 22) = 5.170, *p* = 0.033, η^2^ = 0.190) and group × time effect (F(1, 22) = 28.149, *p* < 0.001, η^2^ = 0.561) (Figure 4).

### 2.9. Effects of the DAZZEON αSleep^®^ Far-Infrared Blanket on Objectively Sleep Quality (Recording via Wearable Devices)

**Figure 5:**
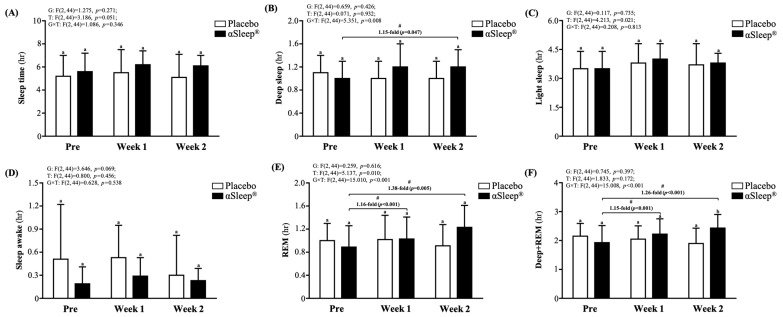
Effects of 2-week DAZZEON αSleep^®^ intervention on (**A**) sleep time, (**B**) deep sleep, (**C**) light sleep, (**D**) awake time, (**E**) REM, and (**F**) deep sleep + REM. Data are expressed as mean ± SD. Different superscript letters (a, b) indicate significant differences at *p* < 0.05 at the same time point. # indicates a significant effect (*p* < 0.05) after 2-week intervention coverage compared with before intervention.

We recorded the subjects’ sleep quality each night using a wearable device and presented this as an average weekly recording (Figure 5). We found that there were no significant differences in weekly sleep time, light sleep time, deep sleep time, wake time, or rapid eye movement (REM) sleep time between the placebo group and the DAZZEON αSleep^®^ group. However, after adding the deep sleep time and REM time, we found that after 14 consecutive days of the intervention, the DAZZEON αSleep^®^ group showed a significant 1.28-fold (F(1, 22) = 6.805, *p* = 0.016) improvement compared with the placebo group. In addition, after one and two weeks of continuous recording, only the REM and REM + deep sleep times of the DAZZEON αSleep^®^ group were significantly increased compared with before the intervention (*p* < 0.05). Light sleep (F(2, 44) = 4.213, *p* = 0.021, η^2^ = 0.161) and REM time (F(2, 44) = 5.137, *p* = 0.010, η^2^ = 0.189) showed significant time effects, and deep sleep (F(2, 44) = 5.351, *p* = 0.008, η^2^ = 0.196), REM (F(2, 44) = 15.010, *p* < 0.001, η^2^ = 0.406), and REM + deep sleep time (F(2, 44) = 15.008, *p* < 0.001, η^2^ = 0.406) showed significant group × time effects.

## 3. Discussion

Currently, the health-promoting application of combining far-infrared ray functions with textiles is still in its early stages. In this study, we found that, after using DAZ for 14 consecutive days, sleep quality significantly improved, which appeared to be related to the significant increase in the concentration of sleep hormones and NO in the blood of the subjects. Furthermore, using DAZZEON αSleep^®^ for 14 consecutive days also enhanced muscle recovery and significantly improved agility. Blood biochemistry and blood cell count analyses were conducted to ensure that the subjects did not experience any adverse reactions or physiological changes during the trial.

A previous study pointed out that FIR radiation demonstrates efficacy in enhancing vascular function and reducing injury in disease models. For instance, FIR exposure activates the promyelocytic leukemia zinc finger protein (PLZF), which mediates protective autophagy in vascular endothelial cells exposed to advanced glycation end products (AGEs), which are common in diabetic vascular injury [11]. Another study reported improvements in vascular access blood flow and reductions in needling pain in hemodialysis patients following FIR therapy, although it did not significantly affect unassisted vascular patency [12]. However, the intensity and distribution of FIR exposure might differ significantly from those of the targeted, medical-grade FIR devices used in other studies [11,12]. A past study on visceral fat’s impact on left ventricular re-modeling via arterial stiffness found a complex interplay between adiposity, vascular compliance, and overall cardiovascular risk, which could be modulated by FIR [13]. Similarly, another study found an association between visceral fat and arterial stiffness, suggesting that systemic factors such as body composition may interact with local thermal therapy effects [14]. In comparison with other studies, we discuss the prognostic value of baPWV on cardiovascular outcomes, which may still contribute to cardiovascular health by maintaining arterial compliance [14,15]. There were no significant inter-group differences in vascular age or arterial stiffness in our study (Table 2). Measurements of vascular age and baPWV may be affected by methodological differences, underlying conditions, and the baseline vascular status of the participants. In addition, this measurement method can only estimate the vascular age and the degree of arteriosclerosis through changes in pulse wave velocity. However, FIR was shown to enhance NO production through increased Ca^2+^ mobilization and the phosphorylation of endothelial nitric oxide synthase (eNOS) at serine 1179 [16]. It was therefore inferred that the ability to promote vasodilation through increased nitric oxide production and improved endothelial function could theoretically reduce arterial stiffness over time [17], which is similar to our findings (Figure 3C). However, the absence of significant changes underscores the need for longer-term studies or those incorporating more comprehensive vascular assessments. Future research should include a broader set of vascular health markers, such as endothelial function tests or dynamic assessments of vascular response to stress, particularly in long-term studies or those focusing on individuals at higher cardiovascular risk.

Previous studies suggest that FIR may help reduce inflammation through mechanisms such as improved blood flow, the modulation of immune function, or direct effects on cytokine expression [18,19]. The potential clinical implications of FIR in reducing inflammatory markers could be emphasized, particularly in the context of managing conditions characterized by chronic inflammation. Reference could be made to similar benefits observed in conditions such as fibromyalgia [20] and Alzheimer’s disease [21,22], suggesting a broader applicability of FIR therapy. One study demonstrated that FIR can ameliorate Pb-induced nephrotoxicity by modulating calcium influx in animal models, suggesting potential systemic effects that might influence blood biochemistry [23]. Similarly, protective effects against ischemia/reperfusion injury have been suggested to act through mechanisms that might involve the modulation of oxidative stress and inflammatory responses [24]. However, our study did not find significant benefits of IL-6 or TNF-α. BDNF levels showed a significant upward trend post-intervention (Figure 3D). This finding is particularly compelling given the emerging literature on the neuroprotective effects of FIR. Studies highlight the potential of FIR to alleviate conditions associated with neuroinflammation and neurodegeneration [22,25]. The increase in BDNF may reflect an enhancement in neuronal survival and function, possibly mediated by improved metabolic profiles and reduced inflammatory markers. However, this study explored the effects of a 2-week intervention using the DAZZEON αSleep^®^ far-infrared blanket on serum levels of melatonin, serotonin, and BDNF. This result is intriguing, as there is limited direct evidence linking FIR exposure to melatonin modulation. Given melatonin’s role in regulating sleep–wake cycles, its increase may corroborate the subjective improvements in sleep quality reported by the participants, although direct mechanisms remain speculative due to the absence of focused studies on FIR’s impact on melatonin synthesis or secretion. A previous study examined FIR’s effects on serotonin levels in depressed patients with insomnia, suggesting that FIR may influence serotonin pathways, contributing to mood regulation and sleep quality [26]. Our findings align with these observations, suggesting that FIR may enhance serotonin synthesis or reduce its degradation, potentially through thermal effects that modify enzymatic activity related to serotonin metabolism. This may contribute to the observed improvements in sleep quality and physical recovery through enhanced tissue oxygenation and nutrient delivery.

In this study, we investigated the impact of the DAZZEON αSleep^®^ blanket infused with FIR technology on subjective sleep quality, as measured by the PSQI. Our results indicate significant improvements in PSQI scores following a two-week intervention period (Figure 4). This suggests a potential therapeutic benefit of FIR-emitting materials in enhancing sleep quality, consistent with prior research examining the physiological effects of FIR on sleep. A previous study observed within-group improvements in sleep quality among subjects wearing FIR-based pajamas, although no significant differences were found between the experimental and sham groups [5]. Similarly, our study showed marked improvements in PSQI scores post-intervention, emphasizing the role of FIR in enhancing sleep among users of the DAZZEON αSleep^®^ blanket. FIR applied to acupoints significantly increased serotonin levels and decreased malondialdehyde levels in depressed patients with insomnia, suggesting a mechanistic pathway through which FIR might improve sleep by modulating biochemical markers associated with sleep and depression [27]. This biochemical pathway might also play a role in our findings, where the FIR technology could be acting through similar physiological mechanisms, thus improving sleep quality. Furthermore, a historical study on the impacts of FIR on rats indicated an increase in slow wave sleep, driven by the modulatory effects of FIR on the circadian rhythm [26]. This evidence supports the hypothesis that FIR could regulate sleep patterns through biological rhythms, potentially explaining the improvements observed in our human study. The integration of FIR technology into everyday sleep environments, such as with the DAZZEON αSleep^®^ blanket, appears to offer a non-invasive method that potentially enhances sleep architecture and overall sleep quality. While our results are promising, further studies with larger sample sizes and diverse populations are necessary to fully establish the efficacy and understand the mechanisms of FIR’s effects on sleep quality. These studies collectively suggest that FIR has a place in the non-pharmacological management of sleep disturbances, particularly in settings where traditional interventions are inadequate or contraindicated.

In addition, we also used Xiaomi Mi Band 7 to obtain objective sleep measurements, capturing total sleep time, deep sleep, light sleep, REM sleep, and wakefulness data. These parameters were measured pre-intervention, at the end of week one, and at the end of week two. In the past, many studies have been conducted on sleep monitoring using wearable devices. Among them, the Xiaomi Mi Band is considered one of tools to objectively assess sleep [28,29]. However, the way in which the Xiaomi Mi Band monitors sleep is mainly by estimating sleep stages through comprehensive analyses of heart rhythm and physical activity monitoring results rather than direct measurement. No significant differences were observed in total sleep time between the placebo and DAZZEON αSleep^®^ groups throughout the study period. Of note is the observation of a slight increase in the durations of deep sleep and REM sleep in the DAZZEON αSleep^®^ group by the end of week two. These stages of sleep are crucial for physical and cognitive restoration. The increases in deep sleep and REM, particularly the significant enhancement in REM sleep from pre- to post-intervention within the DAZZEON αSleep^®^ group, are notable. This stability in wakefulness across both groups indicates that the intervention did not adversely affect sleep continuity. Integrating these findings, the DAZZEON αSleep^®^ far-infrared blanket appears to offer certain physiological benefits during sleep, which may not necessarily translate into subjective perceptions of improved sleep quality (Figure 5). Additionally, an increase in surface temperature after using the DAZZEON αSleep^®^ far-infrared blanket was observed in the current study (Table 2). Previous research has demonstrated that the warm bath effect can shorten sleep latency, extend initial sleep duration, and even promote deeper sleep [30]. This effect may occur by minimizing the gradient between skin temperature and core temperature, thereby reducing energy loss through heat transfer to the environment [31]. Consequently, the combination of an optimal ambient temperature and appropriate bedding is crucial for effective sleep in humans [32]. During sleep, using a blanket or other methods can promote vasodilation (the expansion of blood vessels), which facilitates heat dissipation, lowers core body temperature, and reduces metabolic rate. This, in turn, aids in the release of melatonin, increasing the duration of both deep sleep and REM sleep [33].

In addition to sleep-related research, currently, there is minimal research surrounding the effects of FIR technology on muscle stiffness. Our study utilized the Myoton PRO device, which has been validated in various settings, such as in patients with adhesive capsulitis, where increased muscle stiffness was quantitatively confirmed on the affected sides compared to non-affected sides [34]. The available studies, which explored the impacts of FIR on myofascial neck pain, suggest that, while FIR may decrease muscle stiffness, the effects might not be significant enough to be detected in a healthy, unstressed muscle over a short duration [35]. Further, animal studies, such as those on quadriceps contracture in immobilized rats, primarily focus on pathological or induced conditions where muscle changes are more pronounced and, thus, easier to detect with interventions such as FIR [36]. Our study does not involve a pathological condition that could distinctly alter muscle properties. This observation might explain the lack of noticeable changes in our study, given the short intervention period and the absence of prior muscle stress or injury. However, in our investigation on the effects of the DAZZEON αSleep^®^ intervention over a 2-week period, we observed notable improvements in hand grip strength (Figure 1) and reaction times (Figure 2B). It may be that FIR heat treatment also penetrates deeply into tissue, promoting muscle relaxation and reducing inflammation, which can directly enhance muscle function, as evidenced by the increased grip strength. This finding aligns with that of a study in which far-infrared (FIR) technology demonstrated a positive impact on physical measures, including grip strength, in older adults [37]. However, we used Fitlight as a way to evaluate responsiveness and found significant effects. This may be related to the improvement in blood flow and reduction in muscle stiffness promoted by far-infrared irradiation, which may indirectly enhance neuromuscular coordination and responses. According to a previous report, integrating Fitlight technology into the training regimen significantly enhanced agility and reactivity among junior basketball players. While these training methodologies primarily focused on visual responses and bodily coordination, their positive impacts on overall agility offer valuable insights [38]. These findings suggest that combining far-infrared technology could be an innovative approach within the fields of sports training and rehabilitation, promoting recovery following intense training and potentially enhancing athletic performance in the long term. Future research could explore the specific benefits of various types of far-infrared devices in enhancing athletic capabilities and how this technology could be integrated with traditional training methods to maximize athletes’ performance.

Our research confirms that the use of far-infrared blankets has significant benefits in improving sleep-related hormones, sleep quality, muscle strength, and agility and that it significantly improves vascular age and arteriosclerosis. Safety was also assessed through blood testing. However, this study is still subject to several limitations, including the following: (1) the purpose of this exploratory study was to initially explore a phenomenon, hypothesis, and research area in order to provide a basis for subsequent larger-scale research. Due to the nature of exploratory studies, sample sizes tend to be small, and the focus is on obtaining preliminary data and insights rather than making rigorous statistical inferences. Relevant previous literature has also shown that, under such research conditions, having 12 subjects in each group can meet the acceptability requirements. A larger sample size is needed to strengthen the significance of the results. (2) At present, there are few studies on FIR, so it is difficult to infer a reasonable effective response time. Therefore, we first conducted an evaluation with a short-term intervention of 2 weeks. In the future, further research is needed to explore the benefits or physiological changes brought about by long-term use. (3) The decision to include only men in this study is supported by evidence indicating significant diurnal and circadian variations in core body temperature (CBT) between men and women. Boivin et al. demonstrated that women exhibit a phase advance in the CBT rhythm by approximately 1.1 h compared to men, and their CBT amplitude varies across different phases of the menstrual cycle [39]. These variations can confound the assessment of sleep and physiological responses; hence, to ensure consistency and reduce variability, this study focused on men aged over 45 years. (4) Although far-infrared rays have been developed and applied for a long time, incorporating far-infrared rays into textiles is still novel and rarely studied. (5) Although recording sleep in the same environment and at the same temperature is the best way to reduce variability, we were unable to provide enough space for the subjects to stay in, and we restricted their activity space. In addition, each person’s requirements in terms of sleeping conditions are also very individualized, making it difficult to set consistent restrictions. Therefore, more advanced variable control is needed in the future to meet the research purposes more effectively. Here, we studied everything from cardiovascular and muscle strength to endurance, muscle stiffness, and sleep, and we evaluated biochemical parameters. This is indeed a relatively broad and superficial study; however, it can still serve as a basis for future far-infrared textile applications. Additionally, this study found significant benefits in terms of promoting muscle stiffness and strength, as well as improving sleep. Therefore, more in-depth and complete experiments will be conducted on this issue in the future in order to understand the relevant mechanisms of action.

## 4. Materials and Methods

### 4.1. Study Design and Participants

This study mainly included healthy middle-aged men (over 45 years old) and excluded those with chronic diseases such as hypertension, hyperlipidemia, diabetes, liver disease, kidney disease, gastrointestinal tract disease, cardiovascular disease, cancer, and asthma. Additionally, participants diagnosed with sleep disorders, skin abnormalities, or skin allergies were excluded from the study. This was a double-blind randomized controlled trial involving 24 participants. The study was conducted from 1 May to 31 December 2023. The 24 male participants were randomly divided into two groups, namely, (1) a placebo group and (2) a DAZZEON αSleep^®^ far-infrared blanket group, with 12 participants in each group. The average ages of the participants in the placebo group and the DAZZEON αSleep^®^ group were 53 ± 6 and 54 ± 5 years, respectively. The average heights of the participants in the placebo group and the DAZZEON αSleep^®^ group were 170.9 ± 5.5 and 173.0 ± 4.7 cm, respectively (Table 6).

### 4.2. Intervention

**Figure 6:**
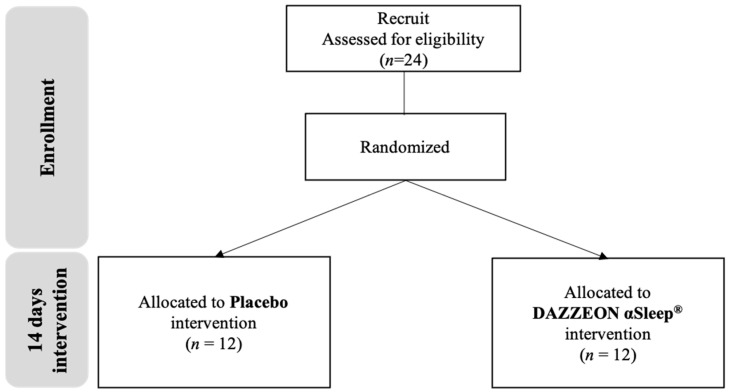
Experimental procedure description.

The participants in both groups were instructed to use their blankets every night for 14 days. The DAZZEON αSleep^®^ group used the DAZZEON αSleep^®^ far-infrared blanket, while the placebo group used an aesthetically similar but inactive blanket. We compared the differences between pre-intervention and post-intervention for 14 consecutive days of use (expressed as pre and post), and we explored the changes before and after a single use and before and after the intervention (Figure 6). In order to avoid the influence of day, night, and time on blood concentration values or body temperature, all our tests were conducted between 09:00 and 12:00 in the morning, and the subjects were required to attend at the same time during the pre-test and post-test.

### 4.3. Sleep Quality

The Xiaomi Mi Band 7 has a built-in three-axis accelerometer, a three-axis gyroscope, a heart rate sensor, and a photoplethysmography sensor for measuring several biomedical parameters. It detects activity status by monitoring the user’s heart rate changes with accelerometer sensors, and it uses built-in algorithms to comprehensively analyze the collected heart rate data and motion data to estimate sleep stages (including total sleep time, deep sleep time (characterized by the lowest heart rate and minimal body movement), light sleep time (detected when there is a moderate heart rate and some body movement), rapid eye movement (REM) sleep time (identified by irregular heart rates and body movements), and wakefulness time). The Xiaomi bracelet connects to the Zepp Life app via Bluetooth, and it transmits and displays the recorded data. During the trial, all subjects were required to wear the Xiaomi Band to sleep every day. Additionally, subjective sleep quality was assessed using the Pittsburgh sleep quality index (PSQI).

### 4.4. Blood Pressure and Oxygen Saturation

Blood pressure was measured using an automatic blood pressure monitor, HEM-1000 (Omron Healthcare Co., Ltd., Kyoto, Japan). Oxygen saturation was measured using a fingertip pulse oximeter (Rossmax SB100, Taipei, Taiwan). These measurements were taken before and after the intervention.

### 4.5. Arterial Stiffness and Vascular Age

Arterial stiffness was assessed using a pulse wave velocity analyzer (Omron HBP-8000, Kyoto, Japan) equipped with four dedicated cuffs. All subjects were asked to lie flat on the bed and place the cuff on their arm with the lower edge ≤ 2.5 cm from the antecubital fossa; they then had to place the cuff on the lower edge of the calf with the lower edge ≤ 2.5~5.0 cm from the medial malleolus. Measurements were taken after 5 min of resting to ensure that they had calmed down, and the ages of the blood vessels were assessed. These measurements were taken before and after the intervention.

### 4.6. Surface Temperature

The subjects were asked to lie flat on the bed, and measurements were taken before and after the intervention and before and after 30 min of blanket use. The researcher was positioned directly above the subject and measured surface temperature using a thermal imaging camera (FLIR E76, Wilsonville, OR, USA).

### 4.7. Muscle Elasticity and Hardness

Muscle elasticity and stiffness were assessed using a Myoton device (Myoton, Tallinn, Estonia). The measurement method used by Myoton PRO involves delivering a mechanical shock at a constant pre-load (0.18 N) to the subcutaneous lipid membrane covering the muscle or tendon being measured. The vibrations of the tissue beneath the probe enable the calculation of the viscoelastic properties of the tissue. We asked the subject to lie flat on the bed, marked the midpoints of the biceps and quadriceps with a marker, and then took the measurement. Measurements were taken before and after the intervention and before and after 30 min of blanket use [34].

### 4.8. Exercise Performance

Handgrip strength: The maximum grip strength of each participant for both hands was evaluated using a Takei digital grip strength meter (T.K.K.5401; Takei Scientific Instruments Co., Ltd., Niigata, Japan). Prior to the main test, the participants were instructed to use minimal force in gripping to ensure a comfortable and standardized grip distance. For the formal test, the researchers randomly selected either the dominant or non-dominant hand to start with. The participants then exerted maximum force while squeezing the gripper with one hand and alternated hands every 60 s to avoid fatigue. This alternating procedure was repeated three times, and the highest grip strength values for both hands were recorded as the data [40].

Maximal oxygen consumption: The 3MISP test was performed to estimate maximal oxygen uptake (VO_2_max) [41]. Initially, the target height for knee elevation was determined by measuring the midpoint between the patella and the iliac crest of each standing participant. This height was marked with colored tape for consistency. The participants wore a Polar H10 Heart Rate Monitor (Polar Electro Oy, Kempele, Finland) with a chest strap to continuously record their heart rate (HR) during the test. The assessment began with the participants stepping in place to the rhythm of an electronic metronome, ensuring each knee lift reached the pre-marked height. The initial step rate was 96 steps per minute (SPM), increasing by 24 SPM each minute for up to three minutes. The participants could transition from walking to running to maintain the rhythm. If a participant could not sustain the required knee height or rhythmic stepping for at least 30 s, the session was terminated, and their data were excluded from the analysis. For safety, a 30 s cooldown period at 80 SPM followed immediately after the test. Heart rate was measured at the start (HR0) and during the first (HR1), second (HR2), and third minutes (HR3) of exercise, as well as during the first minute post-exercise (HR4) [41].

Reaction time test: We used the Fitlight Trainer™ system (FITLIGHT Sports Corporation, Aurora, ON, Canada) for measurement. Four Fitlights were fixed on the wall, two on the left and two on the right, set 80 cm and 180 cm from the ground, respectively, and the distance between the left and right was 100 cm. Four lights lit up randomly and sequentially through the system. The subjects were asked to stand upright in the middle of the light, 100 cm away. After familiarization, a formal test was conducted. When either light came on, we asked the subject to extend their palm forward, covering the illuminated light signals without touching them, and we measured the light every four seconds for a total of 1 min. The average response time to each light signal was calculated through systemic analysis.

These tests were conducted before and after the two-week intervention.

### 4.9. Blood Tests

Blood samples were collected before and after the intervention by a professional nurse. Serum biochemical parameters were analyzed using an automatic biochemical analyzer (Hitachi 7060, Tokyo, Japan), while serum levels of serotonin, melatonin, nitric oxide (NO), interleukin 6 (IL-6), tumor necrosis factor alpha (TNF-α), and brain-derived neurotrophic factor (BDNF) were measured using commercial assay kits. Complete blood count (CBC) was assessed using an automated blood cell analyzer (Sysmex XE-2100, Kobe, Japan).

### 4.10. Statistical Analysis

Between-group comparisons were performed using an unpaired Student’s *t*-test for parametric data and a Mann–Whitney U test for non-parametric data. Differences between the before- and after-intervention measurements were assessed using two-way repeated-measures ANOVA (group × time) or three-way ANOVAs (group × time × measurement) with Bonferroni corrections applied for multiple comparisons. Post hoc analyses were conducted using paired Student’s *t*-tests for parametric data. Statistical significance was set at *p* < 0.05.

## 5. Conclusions

The comprehensive evaluation of the DAZZEON αSleep^®^ far-infrared blanket over a 14-day period revealed a multitude of significant outcomes across various domains. Notably, enhancements in physical performance metrics such as grip strength and reaction ability indicate the blanket’s impact on musculoskeletal function. Moreover, the observed elevations in serotonin, melatonin, and nitric oxide levels suggest favorable modulations of neurotransmitter and vascular signaling pathways. Importantly, subjective reports indicate substantial improvements in sleep quality, as characterized by prolonged durations of deep sleep and REM sleep phases. Collectively, these findings underscore the comprehensive benefits of utilizing the DAZZEON αSleep^®^ far-infrared blanket, positioning it as a promising intervention for promoting cardiovascular health, optimizing physical performance, and enhancing sleep quality.

## Figures and Tables

**Table 1:** Subject’s body composition before and after the 2-week DAZZEON αSleep^®^ intervention.

Body Composition	Pre		Post		ANOVA		
	Placebo	αSleep^®^	Placebo	αSleep^®^	Group	Time	G × T
SpO_2_ (%)	97.6 ± 0.9 ^a^	96.8 ± 1.5 ^a^	97.3 ± 1.1 ^a^	96.8 ± 1.2 ^a^	F(1, 22) = 2.559 *p* = 0.124	F(1, 22) = 0.198 *p* = 0.660	F(1, 22) = 0.551 *p* = 0.466
Body weight (kg)	72.2 ± 5.6 ^a^	77.7 ± 9.4 ^a^	71.8 ± 5.5 ^a^	77.7 ± 9.2 ^a^	F(1, 22) = 3.361 *p* = 0.080	F(1, 22) = 1.985 *p* = 0.173	F(1, 22) = 2.593 *p* = 0.122
BMI (kg/m^2^)	24.7 ± 1.7 ^a^	26.0 ± 3.3 ^a^	24.6 ± 1.6 ^a^	26.0 ± 3.2 ^a^	F(1, 22) = 1.590 *p* = 0.221	F(1, 22) = 1.827 *p* = 0.190	F(1, 22) = 3.020 *p* = 0.096
Muscle mass (kg)	30.8 ± 3.6 ^a^	32.5 ± 3.0 ^a^	30.7 ± 3.5 ^a^	32.5 ± 2.9 ^a^	F(1, 22) = 1.691 *p* = 0.207	F(1, 22) = 0.125 *p* = 0.727	F(1, 22) = 0.499 *p* = 0.488
Fat mass (%)	23.6 ± 5.7 ^a^	25.1 ± 5.6 ^a^	23.4 ± 5.4 ^a^	25.2 ± 5.4 ^a^	F(1, 22) = 0.546 *p* = 0.468	F(1, 22) = 0.071 *p* = 0.792	F(1, 22) = 0.409 *p* = 0.529

Data are presented as mean ± SD. The same superscript letters (a) indicate no significant difference among groups at the same time point.

**Table 2:** Effect of DAZZEON αSleep^®^ on surface temperature before and after the 2-week intervention and before and after the 30 min coverage.

Surface Temperature	Upper Body (°C)		Chest (°C)		Legs (°C)	
	Placebo	αSleep^®^	Placebo	αSleep^®^	Placebo	αSleep^®^
Day 0						
Before	35.7 ± 0.4 ^a^	35.6 ± 0.5 ^a^	35.0 ± 0.7 ^a^	34.9 ± 0.7 ^a^	33.8 ± 0.5 ^a^	34.1 ± 0.5 ^a^
After	36.0 ± 0.4 ^a^	35.9 ± 0.5 ^a^	35.6 ± 0.3 ^a^	35.3 ± 0.6 ^a^	34.7 ± 0.5 ^a^	34.5 ± 0.8 ^a^
Day 14						
Before	35.9 ± 0.5 ^a^	35.7 ± 0.3 ^a^	34.8 ± 0.8 ^a^	34.5 ± 1.1 ^a^	33.9 ± 0.7 ^a^	33.7 ± 0.8 ^a^
After	36.2 ± 0.3 ^a^	36.0 ± 0.3 ^a^	35.5 ± 0.3 ^b^	35.1 ± 0.6 ^a^	34.7 ± 0.6 ^a^	34.1 ± 0.8 ^a^
Group (A)	F(7, 104) = 5.274, *p* = 0.024		F(7, 104) = 2.748, *p* = 0.100		F(7, 104) = 2.829, *p* = 0.096	
Time (B)	F(7, 104) = 3.647, *p* = 0.059		F(7, 104) = 2.933, *p* = 0.090		F(7, 104) = 1.376, *p* = 0.243	
Measurement (C)	F(7, 104) = 13.679, *p <* 0.001		F(7, 104) = 18.861, *p <* 0.001		F(7, 104) = 15.553, *p <* 0.001	
(A) × (B)	F(7, 104) = 0.562, *p* = 0.455		F(7, 104) = 0.083, *p* = 0.774		F(7, 104) = 1.007, *p* = 0.318	
(A) × (C)	F(7, 104) = 0.047, *p* = 0.828		F(7, 104) = 0.204, *p* = 0.652		F(7, 104) = 3.122, *p* = 0.080	
(B) × (C)	F(7, 104) = 0.005, *p* = 0.942		F(7, 104) = 0.415, *p* = 0.521		F(7, 104) = 0.058, *p* = 0.811	
(A) × (B) × (C)	F(7, 104) = 0.015, *p* = 0.904		F(7, 104) < 0.001, *p* = 0.989		F(7, 104) = 0.045, *p* = 0.833	

Data are presented as mean ± SD. Different superscript letters (a, b) indicate significant difference among groups at the same time point (*p* < 0.05).

**Table 3:** Effect of DAZZEON αSleep^®^ on vascular age and level of arteriosclerosis before and after the 2-week intervention and before and after the 30 min coverage.

Arteriosclerosis Examination Instruction	Vascular Age (Years)		Right baPWV (cm/s)		Left baPWV (cm/s)	
	Placebo	αSleep^®^	Placebo	αSleep^®^	Placebo	αSleep^®^
Day 0						
Before	55 ± 11 ^a^	59 ± 11 ^a^	1324 ± 206 ^a^	1313 ± 263 ^a^	1297 ± 234 ^a^	1359 ± 230 ^a^
After	55 ± 11 ^a^	58 ± 9 ^a^	1306 ± 208 ^a^	1285 ± 219 ^a^	1290 ± 232 ^a^	1302 ± 231 ^a^
Day 14						
Before	55 ± 11 ^a^	59 ± 11 ^a^	1311 ± 224 ^a^	1323 ± 212 ^a^	1267 ± 127 ^a^	1363 ± 251 ^a^
After	56 ± 12 ^a^	58 ± 10 ^a^	1263 ± 211 ^b^	1261 ± 195 ^a^	1343 ± 194 ^a^	1234 ± 112 ^a^
Group (A)	F(7, 104) = 0.823, *p* = 0.366		F(7, 104) = 0.008, *p* = 0.930		F(7, 104) = 0.180, *p* = 0.672	
Time (B)	F(7, 104) = 0.116, *p* = 0.734		F(7, 104) = 0.035, *p* = 0.851		F(7, 104) = 0.144, *p* = 0.705	
Measurement (C)	F(7, 104) = 0.105, *p* = 0.746		F(7, 104) = 0.078, *p* = 0.781		F(7, 104) = 0.858, *p* = 0.356	
(A) × (B)	F(7, 104) = 0.020, *p* = 0.875		F(7, 104) = 0.729, *p* = 0.395		F(7, 104) = 0.109, *p* = 0.741	
(A) × (C)	F(7, 104) = 0.153, *p* = 0.942		F(7, 104) = 0.375, *p* = 0.541		F(7, 104) = 0.037, *p* = 0.848	
(B) × (C)	F(7, 104) = 0.116, *p* = 0.734		F(7, 104) = 0.103, *p* = 0.521		F(7, 104) = 0.114, *p* = 0.736	
(A) × (B) × (C)	F(7, 104) < 0.001, *p* = 0.993		F(7, 104) = 0.244, *p* = 0.622		F(7, 104) = 0.072, *p* = 0.789	

Data are presented as mean ± SD. Different superscript letters (a, b) indicate significant difference among groups at the same time point (*p* < 0.05). baPWV, brachial–ankle pulse wave velocity.

**Table 4:** Effect of DAZZEON αSleep^®^ on muscle elasticity and stiffness before and after the 2-week intervention and before and after the 30 min coverage.

Muscle Stiffness Test	Right Biceps Brachii			Left Biceps Brachii			Right Rectus Femoris			Left Rectus Femoris		
	Frequency (Hz)	Stiffness (N/m)	Decrement (N/m)	Frequency (Hz)	Stiffness (N/m)	Decrement	Frequency (Hz)	Stiffness (N/m)	Decrement	Frequency (Hz)	Stiffness (N/m)	Decrement
Day 0—before												
Placebo	15.20 ± 1.35 ^a^	239 ± 27 ^a^	1.50 ± 0.23 ^a^	15.12 ± 1.24 ^a^	240 ± 24 ^a^	1.43 ± 0.32 ^a^	15.93 ± 2.02 ^a^	297 ± 29 ^a^	1.88 ± 0.27 ^a^	15.18 ± 1.50 ^a^	286 ± 25 ^a^	1.95 ± 0.25 ^a^
αSleep^®^	14.91 ± 1.47 ^a^	236 ± 23 ^a^	1.59 ± 0.18 ^a^	14.58 ± 1.57 ^a^	234 ± 18 ^a^	1.47 ± 0.20 ^a^	16.44 ± 1.07 ^a^	308 ± 19 ^a^	1.81 ± 0.22 ^a^	17.52 ± 1.26 ^b^	320 ± 32 ^b^	1.86 ± 0.30 ^a^
Day 0—after												
Placebo	15.09 ± 1.68 ^a^	242 ± 33 ^a^	1.56 ± 0.28 ^a^	15.74 ± 1.42 ^a^	256 ± 29 ^a^	1.45 ± 0.23 ^a^	15.87 ± 1.16 ^a^	302 ± 30 ^a^	1.85 ± 0.40 ^a^	15.64 ± 1.38 ^a^	295 ± 16 ^a^	1.97 ± 0.23 ^b^
αSleep^®^	15.29 ± 1.81 ^a^	246 ± 32 ^a^	1.54 ± 0.23 ^a^	15.14 ± 1.54 ^a^	245 ± 22 ^a^	1.53 ± 0.35 ^a^	16.74 ± 1.05 ^a^	316 ± 21 ^a^	1.76 ± 0.24 ^a^	17.72 ± 1.59 ^b^	317 ± 23 ^b^	1.89 ± 0.41 ^a^
Day 14—before												
Placebo	15.30 ± 1.32 ^a^	244 ± 30 ^a^	1.38 ± 10.19 ^a^	15.49 ± 1.55 ^a^	245 ± 30 ^a^	1.41 ± 0.25 ^a^	15.40 ± 1.39 ^a^	283 ± 19 ^a^	1.86 ± 0.33 ^a^	15.55 ± 1.39 ^a^	284 ± 22 ^a^	1.85 ± 0.33 ^a^
αSleep^®^	14.90 ± 1.53 ^a^	246 ± 26 ^a^	1.47 ± 0.29 ^a^	15.85 ± 1.28 ^a^	254 ± 27 ^a^	1.56 ± 0.41 ^a^	16.74 ± 1.80 ^a^	305 ± 29 ^b^	1.84 ± 0.17 ^a^	15.30 ± 1.18 ^a^	295 ± 27 ^a^	1.79 ± 0.21 ^a^
Day 14—after												
Placebo	14.93 ± 1.58 ^a^	248 ± 25 ^a^	1.61 ± 0.28 ^a^	14.67 ± 1.48 ^a^	235 ± 26 ^a^	1.48 ± 0.24 ^a^	15.50 ± 1.34 ^a^	292 ± 26 ^a^	1.67 ± 0.40 ^a^	15.30 ± 1.18 ^a^	289 ± 22 ^a^	1.83 ± 0.35 ^a^
αSleep^®^	14.64 ± 0.92 ^a^	244 ± 20 ^a^	1.63 ± 0.33 ^a^	15.03 ± 1.13	248 ± 18 ^a^	1.71 ± 0.44 ^a^	16.53 ± 1.18 ^a^	308 ± 25 ^a^	1.70 ± 0.22 ^a^	17.10 ± 1.98 ^b^	305 ± 20 ^a^	1.83 ± 0.21 ^a^
Group (A)	F(7, 104) = 2.750, *p* = 0.100	F(7, 104) = 1.061, *p* = 0.305	F(7, 104) = 0.216, *p* = 0.643	F(7, 104) = 1.319, *p* = 0.253	F(7, 104) = 0.294, *p* = 0.589	F(7, 104) = 2.774, *p* = 0.099	F(7, 104) = 8.552, *p* = 0.004	F(7, 104) = 4.705, *p* = 0.032	F(7, 104) = 0.006, *p* = 0.938	F(7, 104) = 21.505, *p* < 0.001	F(7, 104) = 14.032, *p* < 0.001	F(7, 104) = 2.349, *p* = 0.128
Time (B)	F(7, 104) = 0.686, *p* = 0.409	F(7, 104) = 0.922, *p* = 0.339	F(7, 104) = 0.236, *p* = 0.628	F(7, 104) = 0.041, *p* = 0.839	F(7, 104) = 0.014, *p* = 0.905	F(7, 104) = 0.720, *p* = 0.398	F(7, 104) = 0.792, *p* = 0.376	F(7, 104) = 0.862, *p* = 0.355	F(7, 104) = 1.123, *p* = 0.292	F(7, 104) = 5.357, *p* = 0.023	F(7, 104) = 6.597, *p* = 0.012	F(7, 104) = 2.033, *p* = 0.157
Measurement (C)	F(7, 104) = 0.330, *p* = 0.567	F(7, 104) = 0.124, *p* = 0.725	F(7, 104) = 3.014, *p* = 0.086	F(7, 104) = 0.461, *p* = 0.499	F(7, 104) = 0.320, *p* = 0.573	F(7, 104) = 2.314, *p* = 0.131	F(7, 104) = 0.052, *p* = 0.821	F(7, 104) = 1.683, *p* = 0.197	F(7, 104) = 1.179, *p* = 0.280	F(7, 104) = 1.677, *p* = 0.198	F(7, 104) = 1.569, *p* = 0.213	F(7, 104) = 0.050, *p* = 0.823
(A) × (B)	F(7, 104) = 0.050, *p* = 0.824	F(7, 104) = 0.129, *p* = 0.720	F(7, 104) = 0.001, *p* = 0.981	F(7, 104) = 4.456, *p* = 0.037	F(7, 104) = 5.563, *p* = 0.020	F(7, 104) = 0.854, *p* = 0.358	F(7, 104) = 0.057, *p* = 0.812	F(7, 104) = 0.002, *p* = 0.965	F(7, 104) = 0.734, *p* = 0.394	F(7, 104) = 1.789, *p* = 0.184	F(7, 104) = 1.323, *p* = 0.253	F(7, 104) = 0.605, *p* = 0.438
(A) × (C)	F(7, 104) = 0.610, *p* = 0.437	F(7, 104) = 0.156, *p* = 0.694	F(7, 104) = 0.249, *p* = 0.618	F(7, 104) = 0.056, *p* = 0.814	F(7, 104) = 0.004, *p* = 0.948	F(7, 104) = 0.306, *p* = 0.581	F(7, 104) = 0.178, *p* = 0.674	F(7, 104) = 0.001, *p* = 0.971	F(7, 104) = 0.087, *p* = 0.769	F(7, 104) = 0.736, *p* = 0.393	F(7, 104) = 0.296, *p* = 0.588	F(7, 104) = 0.326, *p* = 0.569
(B) × (C)	F(7, 104) = 0.404, *p* = 0.527	F(7, 104) = 0.190, *p* = 0.663	F(7, 104) = 4.147, *p* = 0.044	F(7, 104) = 4.899, *p* = 0.290	F(7, 104) = 3.686, *p* = 0.058	F(7, 104) = 0.688, *p* = 0.409	F(7, 104) = 0.171, *p* = 0.680	F(7, 104) = 0.042, *p* = 0.838	F(7, 104) = 1.137, *p* = 0.289	F(7, 104) = 0.320, *p* = 0.573	F(7, 104) = 0.246, *p* = 0.621	F(7, 104) = 0.318, *p* = 0.574
(A) × (B) × (C)	F(7, 104) = 0.718, *p* = 0.399	F(7, 104) = 0.595, *p* = 0.442	F(7, 104) = 0.029, *p* = 0.864	F(7, 104) = 0.003, *p* = 0.958	F(7, 104) = 0.128, *p* = 0.721	F(7, 104) = 0.133, *p* = 0.716	F(7, 104) = 0.348, *p* = 0.556	F(7, 104) < 0.001, *p* = 0.987	F(7, 104) = 0.234, *p* = 0.629	F(7, 104) = 2.102, *p* = 0.150	F(7, 104) = 1.033, *p* = 0.312	F(7, 104) = 0.357, *p* = 0.551

Data are presented as mean ± SD. Different superscript letters (a, b) indicate significant differences among groups at the same time point (*p* < 0.05).

**Table 5:** Subjects’ blood biochemical parameters before and after the 2-week DAZZEON αSleep^®^ intervention.

Parameters	Pre		Post		ANOVA		
	Placebo	αSleep^®^	Placebo	αSleep^®^	Group	Time	G × T
Glucose (mg/dL)	94 ± 8 ^a^	99 ± 11 ^a^	94 ± 8 ^a^	98 ± 8 ^a^	F(1, 22) = 1.295 *p* = 0.267	F(1, 22) = 0.014 *p* = 0.908	F(1, 22) = 1.352 *p* = 0.257
AST (U/L)	26 ± 5 ^a^	28 ± 5 ^a^	25 ± 5 ^a^	29 ± 4 ^a^	F(1, 22) = 3.728 *p* = 0.066	F(1, 22) = 0.002 *p* = 0.965	F(1, 22) = 0.240 *p* = 0.629
ALT (U/L)	25 ± 7 ^a^	28 ± 7 ^a^	25 ± 5 ^a^	29 ± 5 ^a^	F(1, 22) = 1.932 *p* = 0.178	F(1, 22) = 0.165 *p* = 0.688	F(1, 22) = 0.225 *p* = 0.640
TC (mg/dL)	210 ± 19 ^a^	212 ± 12 ^a^	211 ± 16 ^a^	213 ± 9 ^a^	F(1, 22) = 0.171 *p* = 0.683	F(1, 22) = 0.535 *p* = 0.472	F(1, 22) = 0.011 *p* = 0.918
TG (mg/dL)	120 ± 28 ^a^	125 ± 31 ^a^	121 ± 26 ^a^	119 ± 31 ^a,^*	F(1, 22) = 0.015 *p* = 0.903	F(1, 22) = 1.913 *p* = 0.181	F(1, 22) = 5.894 *p* = 0.024
HDL (mg/dL)	55.1 ± 6.8 ^a^	50.9 ± 6.5 ^a^	54.7 ± 6.5 ^a^	53.1 ± 6.1 ^a,^*	F(1, 22) = 1.293 *p* = 0.268	F(1, 22) = 3.701 *p* = 0.067	F(1, 22) = 7.538 *p* = 0.012
LDL (mg/dL)	138.6 ± 14.3 ^a^	148.2 ± 9.2 ^a^	139.5 ± 14.4 ^a^	148.2 ± 5.0 ^a^	F(1, 22) = 4.030 *p* = 0.057	F(1, 22) = 0.247 *p* = 0.624	F(1, 22) = 0.247 *p* = 0.624
IL-6 (pg/mL)	1.42 ± 0.32 ^a^	1.28 ± 0.49 ^a^	1.36 ± 0.41 ^a^	1.15 ± 0.52 ^a^	F(1, 22) = 1.509 *p* = 0.232	F(1, 22) = 0.729 *p* = 0.402	F(1, 22) = 0.103 *p* = 0.751
TNF-α (pg/mL)	0.53 ± 0.10 ^a^	0.58 ± 0.11 ^a^	0.58 ± 0.17 ^a^	0.57 ± 0.11 ^a^	F(1, 22) = 0.244 *p* = 0.626	F(1, 22) = 0.310 *p* = 0.583	F(1, 22) = 0.949 *p* = 0.340

Data are presented as mean ± SD. Same superscript letters (a) indicate no significant differences among groups at the same time point. * indicates a significant effect (*p* < 0.05) after 2-week intervention compared with before intervention. AST, aspartate aminotransferase; ALT, alanine transaminase; TC, total cholesterol; TG, triacylglycerol; HDL-C, high-density lipoprotein cholesterol; LDL-C, low-density lipoprotein cholesterol; IL-6, interleukin 6; TNF-α, tumor necrosis factor alpha.

**Table 6:** Basic information.

Basic Information	Placebo	αSleep^®^
Age (year)	54 ± 6 ^a^	54 ± 5 ^a^
Height (cm)	170.9 ± 5.6 ^a^	173.0 ± 4.7 ^a^
Weight (kg)	72.2 ± 5.6 ^a^	77.7 ± 9.4 ^a^

Data are presented as mean ± SD. Same superscript letters (a) indicate no significant differences among groups (*p* > 0.05).

## Data Availability

The data presented in this study are available within the article.

## Supplementary Materials

The following supporting information can be downloaded at: https://www.mdpi.com/article/10.3390/clockssleep6030033/s1, Table S1. Subjects’ blood counts before and after the 2-week DAZZEON αSleep^®^ intervention; Table S2. Subjects’ sleep outcomes before and after the 2-week DAZZEON αSleep^®^ intervention.
