# Supplementary material for: The Impact of DAZZEON αSleep® Far-Infrared Blanket on Sleep, Blood Pressure, Vascular Health, Muscle Function, Inflammation, and Fatigue
Source: Clocks Sleep. 2024 Sep 4;6(3):499–516. doi: 10.3390/clockssleep6030033 (PMC11417803; doi:10.3390/clockssleep6030033)
Supplement: Supplementary file 1 [file clockssleep-06-00033-s001.zip › clockssleep-3173317-supplementary.pdf]

Table S1. Subject's blood count before and after the 2-week DAZZEON αSleep® intervention.

CBC	Pre		Post		*ANOVA*		
	Placebo	αSleep®	Placebo	αSleep®	Group	Time	G×T
WBC (cumm)	5792±799[a]	5792±914[a]	5425±1047[a]	5783±1023[a]	$F(1, 22)=0.237$ $p=0.631$	$F(1, 22)=2.276$ $p=0.146$	$F(1, 22)=2.078$ $p=0.164$
Neutrophils (%)	52.6±8.1[a]	51.6±6.9[a]	52.2±7.9[a]	53.4±6.8[a]	$F(1, 22)<0.001$ $p=0.983$	$F(1, 22)=0.939$ $p=0.343$	$F(1, 22)=2.293$ $p=0.144$
Lymphocytes (%)	36.2±9.5[a]	37.7±8.3[a]	37.0±9.6[a]	36.4±7.2[a]	$F(1, 22)=0.018$ $p=0.894$	$F(1, 22)=0.114$ $p=0.739$	$F(1, 22)=2.771$ $p=0.110$
Monocytes (%)	7.6±1.9[a]	7.2±1.4[a]	7.3±1.3[a]	6.8±1.4[a]	$F(1, 22)=0.527$ $p=0.475$	$F(1, 22)=2.564$ $p=0.124$	$F(1, 22)=0.084$ $p=0.775$
Eosinophils (%)	2.6±1.2[a]	2.9±2.3[a]	2.7±1.3[a]	2.8±1.8[a]	$F(1, 22)=0.114$ $p=0.739$	$F(1, 22)=0.014$ $p=0.906$	$F(1, 22)=0.105$ $p=0.749$
Basophils (%)	1.1±1.2[a]	0.6±0.2[a]	0.9±0.5[a]	0.7±0.2[a]	$F(1, 22)=1.875$ $p=0.185$	$F(1, 22)=0.538$ $p=0.471$	$F(1, 22)=1.081$ $p=0.310$
RBC (MIL/cumm)	4.77±0.25[a]	4.88±0.25[a]	4.78±0.34[a]	4.91±0.30[a]	$F(1, 22)=1.115$ $p=0.303$	$F(1, 22)=0.623$ $p=0.438$	$F(1, 22)=0.117$ $p=0.735$
Hemoglobin (g/dL)	14.4±0.5[a]	14.6±0.8[a]	14.5±0.6[a]	14.8±0.6[a]	$F(1, 22)=1.651$ $p=0.212$	$F(1, 22)=0.661$ $p=0.425$	$F(1, 22)<0.001$ $p=1.000$
Platelets (10^3/uL)	244±40[a]	218±54[a]	241±56[a]	214±47[a]	$F(1, 22)=2.024$ $p=0.169$	$F(1, 22)=0.145$ $p=0.707$	$F(1, 22)=0.009$ $p=0.926$

Data are presented as mean ± SD. Same superscript letters (a) indicate no significant difference among groups at the same time point.

Table S2. Subject's sleep outcomes on pre and post the 2-week DAZZEON αSleep® intervention.

Body composition	Pre		Post		ANOVA		
	Placebo	αSleep®	Placebo	αSleep®	Group	Time	G×T
Bed time (o'clock)	24.0±2.0[a]	23.0±0.9[a]	23.7±2.0[a]	23.1±1.5[a]	$F(1, 22)=1.549$ $p=0.226$	$F(1, 22)=0.279$ $p=0.603$	$F(1, 22)=0.388$ $p=0.775$
Time to fall asleep (min)	16.5±15.7[a]	19.8±16.0[a]	16.3±16.7[a]	20.0±21.5[a]	$F(1, 22)=0.002$ $p=0.553$	$F(1, 22)=1.549$ $p=0.226$	$F(1, 22)<0.001$ $p=1.000$
Wake time (o'clock)	6.0±1.2[a]	6.1±0.7[a]	6.1±1.2[a]	6.4±1.6[a]	$F(1, 22)=0.244$ $p=0.626$	$F(1, 22)=0.644$ $p=0.431$	$F(1, 22)=0.232$ $p=0.635$
Total sleep time (hour)	5.3±1.1[a]	5.9±2.0[a]	5.1±1.4[a]	6.2±1.5[a]	$F(1, 22)=3.382$ $p=0.079$	$F(1, 22)=0.222$ $p=0.882$	$F(1, 22)=0.302$ $p=0.588$

Data are presented as mean ± SD. Same superscript letters (a) indicate no significant difference among groups at the same time point.
